# Impact of the Aortic Geometry on TAVI Prosthesis Positioning Using Self-Expanding Valves

**DOI:** 10.3390/jcm11082259

**Published:** 2022-04-18

**Authors:** Philipp Breitbart, Martin Czerny, Jan Minners, Holger Schröfel, Franz-Josef Neumann, Philipp Ruile

**Affiliations:** 1 Department of Cardiology & Angiology, University Heart Center Freiburg-Bad Krozingen, University Medical Center Freiburg, University of Freiburg, 79189 Bad Krozingen, Germany; jan.minners@uniklinik-freiburg.de (J.M.); franz-josef.neumann@uniklinik-freiburg.de (F.-J.N.); philipp.ruile@uniklinik-freiburg.de (P.R.); 2 Department of Cardiovascular Surgery, University Heart Center Freiburg-Bad Krozingen, University Medical Center Freiburg, University of Freiburg, 79189 Bad Krozingen, Germany; martin.czerny@uniklinik-freiburg.de (M.C.); holger.schroefel@uniklinik-freiburg.de (H.S.)

**Keywords:** TAVI, computed tomography angiography, fusion imaging, THV positioning, aortic geometry, self-expanding valve types

## Abstract

Background: The impact of transcatheter heart valve (THV) position on the occurrence of paravalvular leakage and permanent pacemaker implantation caused by new-onset conduction disturbances is well described. The purpose of this study was to investigate the influence of the geometry of the thoracic aorta on the implantation depth after TAVI (transcatheter heart valve implantation) using self-expanding valve (SEV) types. Methods: We evaluated three-dimensional geometry of the thoracic aorta based on computed tomography angiography (CTA) in 104 subsequently patients receiving TAVI with SEV devices (Evolut R). Prosthesis position was determined using the fusion imaging method of pre- and post-procedural CTA. An implantation depth of ≥4 mm was defined as the cut-off value for low prosthesis position. Results: The mean implantation depth of the THV in the whole cohort was 4.3 ± 3.0 mm below annulus plane. THV position was low in 66 (63.5%) patients and high in 38 (36.5%) patients. After multivariate adjustment none of the aortic geometry characteristics showed an independent influence on the prosthesis position—neither the Sinus of Valsalva area (*p* = 0.335) nor the proximal aortic arch diameter (*p* = 0.754) or the distance from annulus to descending aorta (*p* = 0.309). Conclusion: The geometry of the thoracic aorta showed no influence on the positioning of self-expanding TAVI valve types.

## 1. Introduction

As the number of TAVI procedures continues to grow worldwide, increased knowledge about risk factors for possible complications is crucial for any specific valve design. The impact of the position of the transcatheter heart valve (THV) on the occurrence of paravalvular leakage (PVL) and permanent pacemaker implantation caused by new-onset conduction disturbances (CD) is well described [1].

The fusion imaging method of pre- and post-procedural computed tomography angiography (CTA), published by our group, allows a three-dimensional visualization of the THV within the native annulus plane post TAVI [1]. Using this technique, we confirmed an earlier hypothesis, that an implantation depth of ≥4 mm is a predictor for new CD after TAVI using self-expanding valves (SEV) [2,3].

A reduced calcification of the device landing zone is known as predictor of a low prosthesis position in balloon-expandable valves (BEV) [4]. However, neither the degree of calcification nor other structural annular characteristics are reported to influence the implantation depth using SEV [2]. Although, Gorla et al., identified a correlation between aortic angle and the implantation depth of SEV (Evolut R, Medtronic Inc., Minneapolis, MN, USA) [5].

Therefore, we aimed to examine the influence of the thoracic aorta geometry on the implantation depth after TAVI using SEV devices.

## 2. Methods

### 2.1. Study Population

Candidates for inclusion in this study were all patients with implanted newer generation SEV (Evolut R, Medtronic Inc., Minneapolis, MN, USA) at our institution between January 2015 and June 2020 and an evaluable post-TAVI CTA. As previously described, we perform routine post-TAVI CTA in all eligible patients in our institution [1]. Exclusion criteria were poor image quality, which does not allow fusion imaging, valve-in-valve procedures or the need for peri-interventional conversion to surgical treatment.

A first sub-analysis of these patients was recently published, examining the influence of SEV prosthesis position on the occurrence of TAVI complications [2]. All TAVI procedures were performed by experienced operators (each with an experience of at least 100 TAVI-procedures) via a transfemoral access. The study was approved by the local institutional review board (IRB number EF FR 472/12, University of Freiburg, Freiburg, Germany) and complies with the Declaration of Helsinki. All patients gave written informed consent for TAVI and the anonymized use of clinical, procedural and follow-up data at the time of the intervention.

### 2.2. Image Acquisition

We used a second generation dual-source CT scanner (Somatom Definition Flash, Siemens Healthineers, Forchheim, Germany) for the retrospective ECG-gated contrast-enhanced pre- and post-TAVI CTAs (70 mL for pre- and 50 mL for post-TAVI CTA, Imeron 400, Bracco, Konstanz, Germany). The detailed CTA-protocol was published previously [1]. All patients received the post-TAVI CTAs pre-discharge. Two experienced readers (P.B. and P.R.) conducted the image analysis in consensus using two post-processing workstations (Syngo Multimodality Workplace, Siemens Healthineers, Forchheim, Germany and 3mensio Structural Heart, Pie Medical Imaging, Maastricht, The Netherlands).

### 2.3. Image Analysis

All detailed measurements of the device landing zone and the process of fusion imaging were described previously [1]. The fused pre- and post-procedural CTA images were used for an assessment of the final prosthesis position. The implantation depth, defined as THV length below the annular plane, was measured separately for all three cusps: left coronary cusp (LCC), right coronary cusp (RCC) and non-coronary cusp (NCC). An implantation depth of ≥4 mm was defined as cut-off value for a low prosthesis position.

### 2.4. Assessment of Aortic Geometry

**Figure 1:**
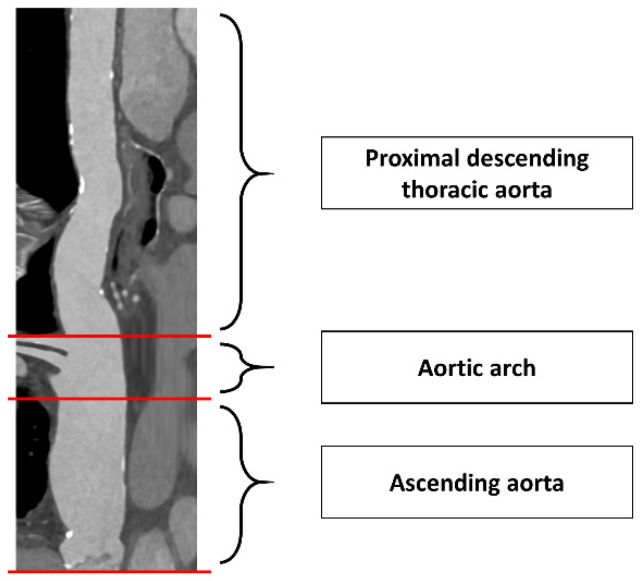
Division of the thoracic aorta. Pre-TAVI CTA “stretched vessel” reconstruction for division of the thoracic aorta into three sections: (1) Ascending aorta (annular plane to proximal origin of brachiocephalic artery); (2) Aortic arch (proximal origin of brachiocephalic artery to distal origin of left subclavian artery); (3) Proximal descending thoracic aorta (distal aortic arch end to the plane at transverse level of the annular plane).

First, a centerline was created from the annular plane to the comparable height within the descending aorta. According to Rylski et al., we divided the thoracic aorta into three sections by appropriate planes perpendicular to the centerline (Figure 1): (1) Ascending aorta from the annular plane to the proximal origin of the brachiocephalic artery; (2) Aortic arch from the proximal origin of the brachiocephalic artery ranging up to the distal end of the left subclavian artery origin; (3) Proximal descending thoracic aorta from the end of the aortic arch until the plane at the transverse level of the annular plane [6].

Planimetric measurements of the area (including area derived diameter) and the maximum/minimum diameter were conducted at the following planes perpendicular to the centerlines: Sinus of Valsalva, the maximum ascending aorta, the planes immediately proximal to the brachiocephalic artery origin (= proximal aortic arch) as well as distal to the left carotid (= middle aortic arch) and the left subclavian artery origin (= distal aortic arch), and finally descending aorta at the transverse level of the annular plane [6].

The length of ascending aorta (L) was measured following the centerline from annular plane till the proximal aortic arch plane immediately proximal the brachiocephalic artery origin [6]. Additionally, we determined the incremental curve length of the aorta from the annular plane to the middle and distal aortic arch.

**Figure 2:**
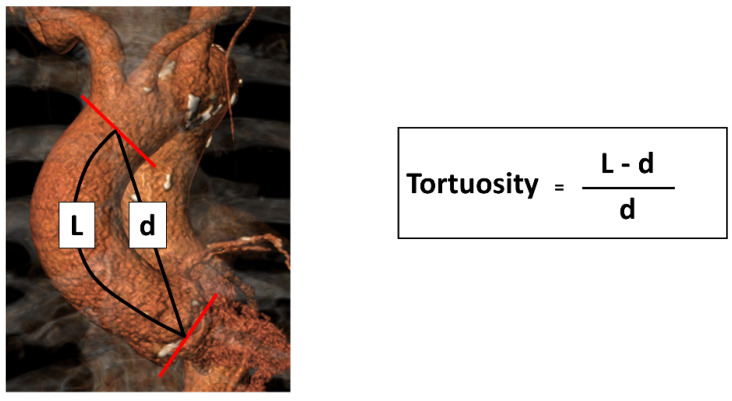
Pre-TAVI CTA in Volume Rendering Technique (VRT) for determination of Tortuosity. Tortuosity of the ascending aorta was calculated as ratio of its curved length (= L, curved black line) to the linear distance (= d, straight black line) between its two endpoints (annular plane and the proximal origin of the brachiocephalic artery, each marked with the red lines).

Tortuosity of the ascending aorta was calculated as ratio of its length (L) to the linear distance (d) between their two endpoints (annular plane and the proximal origin of the brachiocephalic artery) (Figure 2).

**Figure 3:**
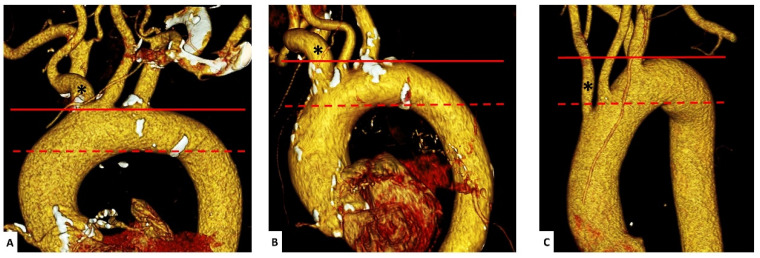
Types of the aortic arch. Pre-TAVI CTA in Volume Rendering Technique (VRT) for evaluation of the aortic arch. The types were defined as I (origin of the brachiocephalic artery at the height of the maximum cranial curvature of the aortic arch; (**A**), Type II (origin between the maximum cranial and caudal curvature; (**B**) and Type III (origin below the caudal curvature; (**C**). The maximum cranial (-----) and maximum caudal (-) curvature of the aortic arch as well as the brachiocephalic artery (*) are delineated.

We defined the type of the aortic arch as Type I (origin of the brachiocephalic artery at the height of the maximum cranial curvature of the aortic arch), Type II (origin between the maximum cranial and caudal curvature) and Type III (origin below the caudal curvature) (Figure 3) [7].

**Figure 4:**
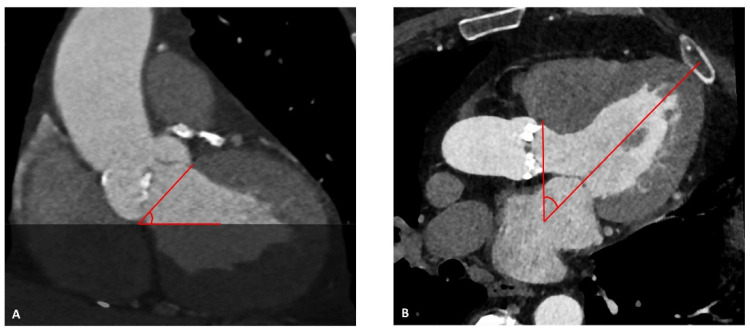
Assessment of aortic and annular angle. Pre-TAVI CTA reconstructions for measurement of the aortic angle between the annular and ideal horizontal plane (**A**) and the annular angle between the annular plane and the longitudinal axis of the left ventricle (**B**).

The aortic angle was assessed as the angle between annular plane and ideal horizontal plane (Figure 4A) [5]. Additionally, we identified the annular angle between the annular plane and the longitudinal axis of the left ventricle (Figure 4B).

The angle of ascending aortic curvature is defined as the angle between the perpendicular line to the annular plane and the perpendicular line to the cross-sectional plane of the aorta at the level of the brachiocephalic artery [8,9].

**Figure 5:**
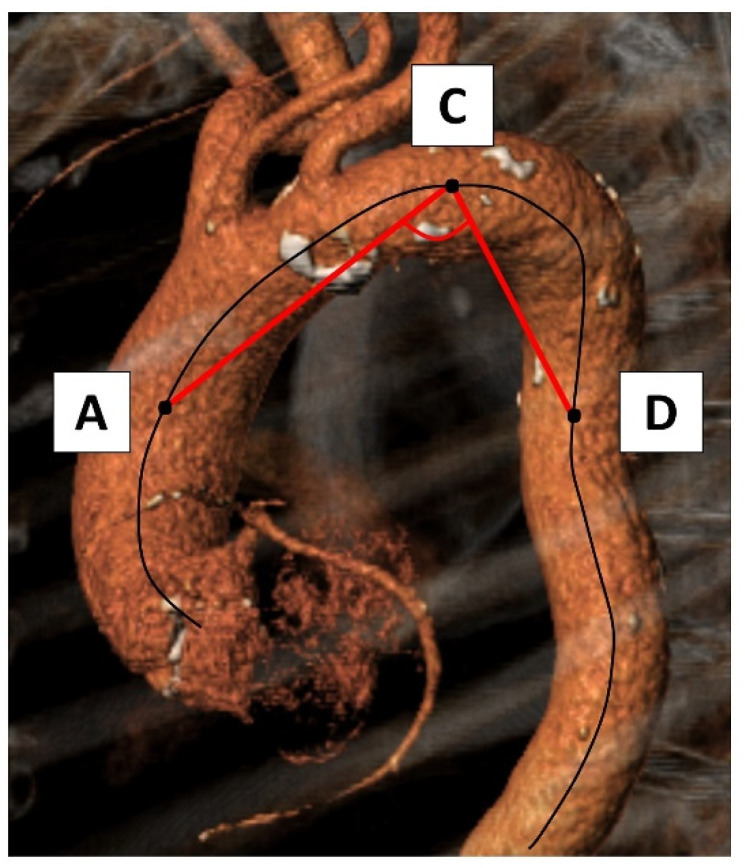
Evaluation of the aortic apex angle. For assessment of the aortic apex angle the center points of the ascending (A) and descending aorta (D) at the mid-level of the right pulmonary artery flow were defined as well as the most cranial point (C) on the centerline (= curved black line) in the aortic arch expressing the apex of the arch. The angle between the lines CA and CD expresses the aortic apex angle.

For the aortic apex angle we first determined the center points of the ascending (A) and descending aorta (D) at the mid-level of the right pulmonary artery flow [10,11]. The third landmark was placed on the centerline of the most cranial point (C) in the aortic arch—called the apex of the arch. The angle between the lines CA and CD expresses the aortic apex angle (Figure 5).

Additionally, we measured the axial distance between A and D (= ascending descending distance) and between the central points of the annular plane and the descending aorta at the transverse level of the annular plane (= annulus descending distance).

### 2.5. Statistical Analysis

Statistical analysis was performed using SPSS software, Version 25.0 (IBM Corp., Armonk, NY, USA) and MedCalc, Version 19.4 (MedCalc Software Ltd., Ostend, Belgium). We used the χ2-test (for categorical variables), the Student’s *t*-test (for normal distributed continuous variables) or the Mann–Whitney-U Test (non-normal distributed continuous variables) to test differences between the groups with high and low THV position. Univariate and multivariate logistic regression models were performed to assess possible predictors for a low prosthesis position. All variables with a *p*-value < 0.05 in the group comparison were included in the univariate and—if significant—in the multivariate logistic regression models. When multiple variables were directly related (e.g., Sinus of Valsalva area and Sinus of Valsalva maximum diameter), we chose the variable with the lowest *p*-value. A two-sided *p*-value of <0.05 was defined as statistically significant in all tests.

## 3. Results

In total, 118 consecutive patients received newer generation SEV and post-TAVI CTA within the study period. The image quality of post-TAVI CTAs was too poor in 2 patients; 12 further patients were excluded due to a valve-in-valve procedure or a surgical revision caused by a THV dislocation. Finally, we included 104 patients. Details of the study population were described elsewhere [2]. The mean age of the study cohort (66.3% female) was 82.2 ± 5.2 years with a mean logistic Euroscore of 15.1 ± 11.3%. The mean implantation depth of the THV in the whole cohort was 4.3 ± 3.0 mm below the annulus plane. The baseline, procedural and prosthesis-related characteristics are presented in Table 1.

### 3.1. Aortic Geometry and Prosthesis Position

Using an implantation depth of ≥4 mm as cut-off value, THV position was low in 66 (63.5%) patients and high in 38 (36.5%) patients. All aortic geometry characteristics are presented in Table 2. Patients with a low THV position showed larger maximum diameters of the Sinus of Valsalva (33.4 ± 3.3 mm vs. 31.7 ± 3.4 mm, *p* = 0.013), ascending aorta (36.4 ± 3.9 mm vs. 34.5 ± 4.2 mm, *p* = 0.024) and proximal aortic arch (33.9 ± 3.3 mm vs. 32.6 ± 2.9 mm, *p* = 0.035). Additionally, we found a longer distance from annulus to descending aorta (83.0[72.8;92.3] mm vs. 76.5[71.0;81.8] mm, *p* = 0.033) and longer distance from ascending to descending aorta (99.0 ± 12.7 mm vs. 93.7 ± 11.9 mm, *p* = 0.037) in this group.

Most patients showed an aortic arch type II (78.8%) without any influence on the implantation depth (*p* = 0.668). Furthermore, the aortic angle (*p* = 0.733), length of ascending aorta (*p* = 0.150) or aortic apex arch angle (*p* = 0.334) were similar between patients with high and low prosthesis position.

### 3.2. Logistic Regression Analysis

We included the Sinus of Valsalva area, maximum diameter of ascending aorta, mean diameter of proximal aortic arch, as well as the annulus and ascending descending distance in the logistic regression models. After multivariate adjustment none of the aortic geometry characteristics showed an independent influence on the prosthesis position (Table 3).

## 4. Discussion

In this investigation we examined the influence of aortic geometry on positioning of the self-expanding Evolut R TAVI prostheses using 3D fusion imaging of pre- and post-TAVI CTA. Our results demonstrate that none of aortic geometric features is associated with a low prosthesis position, possibly generating new conduction disturbances. Hence, a detailed assessment of the aortic anatomy seems not to be mandatory before implantation of these prostheses.

While the association between THV implantation depth and the occurrence of TAVI complications is well described [1,2,12,13], information on predictors of low prosthesis positioning using SEV devices is scarce. An accurate assessment of the geometry of the thoracic aorta is crucial in cardiac surgery and some characteristics have been identified as predictors of dissections [6,8,14]. A change in aortic geometry with aging is also described [10].

Up to now, only the impact of aortic angle on THV position was assessed [5]. Contrary to our results, Gorla et al., described a low implantation depth after using Evolut R prosthesis in patients with horizontal aorta (aortic angle > 57°) [5]. However, in this trial the THV position was only assessed in two-dimensional angiographic images and the relationship between implantation depth and aortic angle was evaluated solely on the basis of the cut-off value and not linearly.

To the best of our knowledge, we are the first group to investigate the influence of the complex geometry of the thoracic aorta on THV using SEV. Using fusion imaging with its 3D capacity we were able to demonstrate that THV positioning in SEV is unaffected by specific aortic geometric features. Therefore, we conclude that a separate assessment of aortic geometry is not crucial when planning the procedure in SEV.

## 5. Limitations

The limited sample size of our cohort limits the power to identify minor anatomic predictors for a low prosthesis position. Nevertheless, the statistical analysis seems sufficient to conclude that the aortic geometry has no major influence on the THV implantation depth. Moreover, our study was limited to patients with one specific SEV type. It remains speculative, if these results are transferable to other SEV types or BEV.

## 6. Conclusions

The geometry of the thoracic aorta showed no influence on the positioning of self-expanding TAVI valve types.

## 7. Impact on Daily Practice

Prevention of a low prosthesis position after TAVI is desirable to reduce the occurrence of new conduction disturbances. The results of our current study imply that a separate analysis of the thoracic aortic geometry is not mandatory when planning the TAVI-procedure using SEV.

## Figures and Tables

**Table 1:** Baseline, procedural and prosthesis-related characteristics of the entire study population.

		All Patients (*n* = 104)
Age	(years)	82.2 ± 5.2
Female		69 (66.3)
BMI	(kg/m^2^)	27.8 ± 4.9
Logistic Euroscore	(%)	15.1 ± 11.3
Preexisting conduction disturbances	Pacemaker	5 (4.8)
	Total Conduction disturbances	48 (46.2)
Atrial fibrillation		29 (27.9)
Aortic valve area	(cm^2^)	0.72 ± 0.22
Aortic valve type	Tricuspid	98 (94.2)
	Bicuspid	6 (5.8)
Annulus diameter	(mm)	23.1 ± 2.3
Grade of calcification of the device landing zone	total	4.2 ± 1.1
	Left coronary cusp	1.4 ± 0.5
	Right coronary cusp	1.3 ± 0.5
	Non-coronary cusp	1.5 ± 0.5
Ejection fraction pre-interventional	(%)	50.6 ± 10.3
Access route	Transfemoral	103 (99.0)
	Trans-subclavian	1 (1.0)
Prosthesis size	23 mm	5 (4.8)
	26 mm	46 (44.2)
	29 mm	42 (40.4)
	34 mm	11 (10.6)
	Mean	4.3 ± 3.0
Implantation depth below	Left coronary cusp	4.9 ± 2.8
annulus (mm)	Right coronary cusp	4.9 ± 3.4
	Non-coronary cusp	3.1 ± 3.5

Values are mean ± standard deviation or *n* (%). BMI: body mass index.

**Table 2:** Aortic geometry characteristics of the entire study population and in patients with a high and low THV position.

		All Patients (*n* = 104)	High Position (*n* = 38)	Low Position (*n* = 66)	*p*-Value
Aortic angle	(°)	49.5 [44.3; 54.8]	48.5 [42.8; 54.3]	50.0 [45.0; 55.0]	0.733
Annular angle	(°)	111.0 [103.0; 123.0]	109.5 [103.0; 123.3]	111.5 [103.0; 120.3]	0.927
Sinus of Valsalva area	(mm^2^)	721.0 [612.3; 816.3]	645.5 [571.8; 776.3]	756.5 [658.0; 825.5]	0.005
Sinus of Valsalva maximum diameter	(mm)	32.8 ± 3.5	31.7 ± 3.4	33.4 ± 3.3	0.013
Sinus of Valsalva minimum diameter	(mm)	27.9 ± 3.0	26.9 ± 3.0	28.5 ± 2.9	0.010
Ascending aorta area	(mm^2^)	953.1 ± 234.6	896.4 ± 254.3	985.8 ± 217.8	0.061
Ascending aorta mean diameter	(mm)	34.7 ± 4.1	33.6 ± 4.2	35.3 ± 3.8	0.040
Ascending aorta maximum diameter	(mm)	35.7 ± 4.1	34.5 ± 4.2	36.4 ± 3.9	0.024
Ascending aorta minimum diameter	(mm)	33.6 ± 4.0	32.7 ± 4.3	34.1 ± 3.8	0.076
Proximal aortic arch area	(mm^2^)	804.6 ± 153.7	769.3 ± 143.1	825.0 ± 157.0	0.075
Proximal aortic arch mean diameter	(mm)	32.0 ± 3.0	31.1 ± 2.8	32.5 ± 3.0	0.024
Proximal aortic arch maximum diameter	(mm)	33.4 ± 3.2	32.6 ± 2.9	33.9 ± 3.3	0.035
Proximal aortic arch minimum diameter	(mm)	30.6 ± 2.9	30.0 ± 2.7	30.9 ± 3.0	0.118
Type of the aortic arch					0.668
I	n	15 (14.4)	4 (10.5)	11 (16.7)	
II	n	82 (78.8)	31 (81.6)	51 (77.3)	
III	n	7 (6.7)	3 (7.9)	4 (6.1)	
Middle aortic arch area	(mm^2^)	591.6 ± 113.3	569.5 ± 116.9	604.3 ± 110.1	0.133
Middle aortic arch mean diameter	(mm)	27.4 ± 2.5	26.9 ± 2.5	27.7 ± 2.5	0.134
Middle aortic arch maximum diameter	(mm)	28.9 ± 2.7	28.4 ± 2.7	29.2 ± 2.7	0.159
Middle aortic arch minimum diameter	(mm)	25.7 ± 2.6	25.2 ± 2.7	26.0 ± 2.5	0.114
Distal aortic arch area	(mm^2^)	500.8 ± 93.7	481.2 ± 82.0	512.1 ± 98.7	0.105
Distal aortic arch mean diameter	(mm)	25.2 ± 2.3	24.7 ± 2.2	25.4 ± 2.4	0.123
Distal aortic arch maximum diameter	(mm)	26.4 ± 2.5	26.0 ± 2.4	26.6 ± 2.5	0.221
Distal aortic arch minimum diameter	(mm)	24.0 ± 2.2	23.5 ± 1.9	24.3 ± 2.3	0.090
Descending aorta area	(mm^2^)	445.4 ± 94.8	425.4 ± 68.7	456.9 ± 105.8	0.103
Descending aorta area mean diameter	(mm)	23.8 ± 2.7	23.3 ± 1.9	24.2 ± 3.1	0.131
Descending aorta area maximum diameter	(mm)	24.9 ± 2.6	24.3 ± 1.8	25.2 ± 2.9	0.087
Descending aorta area minimum diameter	(mm)	22.7 ± 2.4	22.4 ± 2.0	22.9 ± 2.6	0.296
Length of ascending aorta	(mm)	91.2 ± 10.3	89.3 ± 10.2	92.3 ± 10.4	0.150
Tortuosity		0.19 [0.13; 0.24]	0.20 [0.13; 0.26]	0.18 [0.13; 0.24]	0.541
Aortic distance till middle aortic arch	(mm)	111.1 ± 12.2	108.1 ± 11.0	112.8 ± 12.6	0.060
Aortic distance till distal aortic arch	(mm)	126.5 [118.0; 138.0]	123.5 [116.0; 131.0]	127.0 [119.0; 139.0]	0.141
Angle of ascending aortic curvature	(°)	93.0 [85.3; 103.0]	92.0 [84.5; 105.0]	93.0 [85.8; 102.0]	0.885
Aortic apex arch	(°)	89.2 ± 10.6	87.9 ± 10.1	89.9 ± 10.9	0.334
Distance from annulus to descending aorta	(mm)	80.0 [72.0; 90.0]	76.5 [71.0; 81.8]	83.0 [72.8; 92.3]	0.033
Distance from ascending to descending aorta	(mm)	97.1 ± 12.6	93.7 ± 11.9	99.0 ± 12.7	0.037

**Table 3:** Univariate and multivariate logistic regression model analysis of predictors for a low prosthesis position after TAVI.

	Univariate		Multivariate	
	Odds Ratio [95% CI]	*p*-Value	Odds Ratio [95% CI]	*p*-Value
Sinus of Valsalva area	1.004 [1.001–1.007]	0.015	1.002 [0.998–1.006]	0.335
Ascending aorta maximum diameter	1.138 [1.014–1.277]	0.028	1.048 [0.870–1.262]	0.620
Proximal aortic arch mean diameter	1.187 [1.018–1.384]	0.028	1.041 [0.810–1.338]	0.754
Distance from annulus to descending aorta	1.041 [1.002–1.082]	0.037	1.030 [0.973–1.089]	0.309
Distance from ascending to descending aorta	1.039 [1.002–1.078]	0.041	0.997 [0.941–1.057]	0.927

## Data Availability

Transferring data to third parties is not included in the ethics statement. Please contact the corresponding authors for submitting a request to the ethics committee, if desired.

## Abbreviations

BEV	Balloon-expandable valves
CD	Conduction disturbances
CTA	Computed tomography angiography
ECG	Electrocardiogram
LVOT	Left ventricular outflow tract
PVL	Paravalvular leakage
SEV	Self-expanding valves
TAVI	Transcatheter aortic valve implantation
THV	Transcatheter heart valve
